# Crosstalk between *Helicobacter pylori* and Gastric Epithelial Cells Is Impaired by Docosahexaenoic Acid

**DOI:** 10.1371/journal.pone.0060657

**Published:** 2013-04-05

**Authors:** Marta Correia, Valérie Michel, Hugo Osório, Meriem El Ghachi, Mathilde Bonis, Ivo G. Boneca, Hilde De Reuse, António A. Matos, Pascal Lenormand, Raquel Seruca, Ceu Figueiredo, Jose Carlos Machado, Eliette Touati

**Affiliations:** 1 Institute of Molecular Pathology and Immunology of the University of Porto (IPATIMUP), Rua Dr. Roberto Frias s/n, Porto, Portugal; 2 Faculdade de Medicina da Universidade do Porto, Al. Hernani Monteiro, Porto, Portugal; 3 Institut Pasteur, Unité de Pathogenèse de Helicobacter, Paris, France; 4 Institut Pasteur, G5 Biologie et Génétique de la Paroi Bactérienne, Paris, France; 5 INSERM, Group AVENIR, 75724 Paris, France; 6 Anatomia Patológica, Centro Hospitalar de Lisboa Central, Lisboa, Portugal; 7 Centro de Estudos do Ambiente e do Mar (CESAM/FCUL) – Faculdade de Ciências da Universidade de Lisboa and Centro de Investigação Interdisciplinar Egas Moniz (CiiEM) Campo Grande, Lisboa, Portugal; 8 Platform of Proteomic, Institut Pasteur, Paris, France; University of Aberdeen, United Kingdom

## Abstract

*H. pylori* colonizes half of the world's population leading to gastritis, ulcers and gastric cancer. *H. pylori* strains resistant to antibiotics are increasing which raises the need for alternative therapeutic approaches. Docosahexaenoic acid (DHA) has been shown to decrease *H. pylori* growth and its associated-inflammation through mechanisms poorly characterized. We aimed to explore DHA action on *H. pylori-*mediated inflammation and adhesion to gastric epithelial cells (AGS) and also to identify bacterial structures affected by DHA. *H. pylori* growth and metabolism was assessed in liquid cultures. Bacterial adhesion to AGS cells was visualized by transmission electron microscopy and quantified by an Enzyme Linked Immunosorbent Assay. Inflammatory proteins were assessed by immunoblotting in infected AGS cells, previously treated with DHA. Bacterial total and outer membrane protein composition was analyzed by 2-dimensional gel electrophoresis. Concentrations of 100 µM of DHA decreased *H. pylori* growth, whereas concentrations higher than 250 µM irreversibly inhibited bacteria survival. DHA reduced ATP production and adhesion to AGS cells. AGS cells infected with DHA pre-treated *H. pylori* showed a 3-fold reduction in Interleukin-8 (IL-8) production and a decrease of COX2 and iNOS. 2D electrophoresis analysis revealed that DHA changed the expression of *H. pylori* outer membrane proteins associated with stress response and metabolism and modified bacterial lipopolysaccharide phenotype. As conclusions our results show that DHA anti-*H. pylori* effects are associated with changes of bacteria morphology and metabolism, and with alteration of outer membrane proteins composition, that ultimately reduce the adhesion of bacteria and the burden of *H. pylori*-related inflammation.

## Introduction

*H. pylori* infection affects half of the world population and is associated with chronic gastritis, ulcer disease and gastric cancer [1]. Eradication of *H. pylori* infection, ideally before gastric injuries might prevent the development of atrophy and precancerous lesions [2]–[4]. Standard recommended treatment to eradicate *H. pylori* infection consists in an association of two antibiotics, usually amoxicillin with clarithromycin, or metronidazole, with a proton pump inhibitor [5]. The efficacy of this prescription has decreased over time, and currently holds less than 80% of success, mainly due to an increase incidence of *H. pylori* strains resistant to clarithromycin [6], [7].

*H. pylori* chronic infection is characterized by an inflammation of the gastric mucosa that varies in severity according to the strain characteristics, host susceptibility genes and diet [8], [9]. It is well demonstrated that gastric epithelial cells respond to *H. pylori* infection by up-regulating the expression of pro-inflammatory genes, including cyclooxygenase-2 (COX2), inducible nitric oxide synthase (iNOS), and interleukin(IL)-8 [10]–[12]. IL-8 plays an important role in the chemoattraction of neutrophils to inflammation sites and their further activation. Alternative therapeutic approaches and new treatment strategies that can overcome *H. pylori* resistance strains to antibiotics are of great interest. The antimicrobial activity of certain non-antibiotic compounds has been addressed and deserves further attention. In agreement with a previous study [13], we have demonstrated that Docosahexaenoic acid (DHA), an n-3 polyunsaturated fatty acid (n-3 PUFA), known for its anti-inflammatory action [14]–[16], causes an anti-*H. pylori* growth effect and decreases gastric inflammation in mice infected by *H. pylori* [13], [17]. The mechanisms through which DHA exerts its effects are not well characterized. We postulate that DHA's bacteriostatic effect involves changes in the bacterium cell envelop.

In the present study, we aimed to identify bacterial structures impaired by DHA and to understand how DHA counteracts *H. pylori-*associated inflammation. For this purpose we analyzed the effects of DHA on *H. pylori* outer membrane protein composition and on the ability of bacteria to adhere to host gastric epithelial cells and on the associated inflammation. Our results demonstrate that DHA inhibits *H. pylori* growth through alteration of bacterial membrane protein composition leading to impaired bacteria-host cell adhesion and a reduced burden of the bacteria-related inflammation.

## Methods

### Fatty acids, *H. pylori* strains and culture conditions

DHA was obtained from *Cayman Chemical Company* (Michigan, USA) with a degree of purity of 99% in ethanol. The *H. pylori* strains used in this study were: SS1 [18], B128 [19] and 26695 (ATCC 700392) (ATCC, Rockville, MD). *H. pylori* was grown on blood agar base 2 plates (Oxoid, Lyon, France) supplemented with 10% defibrinated horse blood (bioMérieux, Marcy l'Etoile, France). Plates were incubated at 37°C for 48 h to 72 h under microaerobic conditions (7% O_2_, 10% CO_2_; anoxomat system). To determine growth kinetics, plate-grown *H. pylori* strains were inoculated to an initial optical density of 0.03 at 600 nm into liquid Brucella broth (BB) (Oxoid) supplemented with 10% fetal calf serum (FCS).

### DHA treatment of *H. pylori* cultures

To establish *H. pylori* growth curves, 18–20 h bacteria cultures were diluted in 10 ml of medium with or without DHA to an initial OD600 of 0.03. To assess *H. pylori* inhibitory growth effect, DHA was added to the liquid culture after 12 h of growth. Each experiment, consisting of a control (non-DHA treated *H. pylori* liquid culture) and DHA treated conditions (*H. pylori* liquid culture incubated with 50 µM, 100 µM, 250 µM and 500 µM of DHA) was performed in triplicate. Every 12 h the OD600 of liquid cultures was measured and aliquots were serially diluted and plated on blood agar plates to determine the number of viable bacteria by counting the number of colony forming units (CFU). To assess the reversibility of the DHA inhibitory effect on *H. pylori* growth, bacteria previously incubated for 24 h with increasing concentrations of DHA from 50 µM to 250 µM were washed, then resuspended and cultured in fresh medium.

### ATP assay

The metabolic activity of bacteria was determined by the measurement of their ATP production over 24 h using the BacTiter-Glo™ microbial cell viability test according to supplier's instruction (Promega). *H. pylori* strain 26695 was grown in BB with increasing concentrations of DHA, as previously described. The relative amount of ATP was quantified on culture samples every 6 hours. The luminescence detected was proportional to the amount of ATP expressed per 10^6^ bacteria per OD units.

### Analysis of *H. pylori* adherence to gastric epithelial cells

To assess the interaction of *H. pylori* to gastric epithelial cells (AGS: gastric adenocarcinoma, CRL-1739, ATCC-LGC®), transmission electron microscopy (TEM) analysis was performed. Briefly, 2.0×10^4^ AGS cells were grown in RPMI medium 1640 supplemented with 10% FCS and 1% of penicillin-streptomycin in 96 well plates, for 48 h at 37°C until confluence. Liquid *H. pylori* cultures pre-treated with 50 µM and 100 µM of DHA during 24 h were added to cells at a multiplicity of infection (MOI) of 500∶1, at 37° C with shaking for 3 h. Cells were then fixed in glutaraldehyde-3%/sodium cacodylate (pH 7.3; 0.1 M) overnight at 4°C until further TEM analysis.

To analyze the effect of DHA on the *H. pylori* ability to adhere to gastric epithelial cells, 2.0×10^4^ AGS cells were grown until reaching 100% confluence. Liquid *H. pylori* cultures, pretreated with 50 µM, 100 µM and 250 µM of DHA during 24 h were added to AGS cells at MOI of 100∶1 at 37° C with shaking for 30 minutes. AGS cells were incubated for 30 minutes with 1% PBS-BSA and for 1 h with a rabbit polyclonal anti-*Helicobacter* antibody (USB, USA) and revealed by enzyme-linked immunosorbent assay (ELISA) [20].

### DHA effect on the inflammatory response of gastric epithelial cells infected by *H. pylori*

IL-8 production of AGS cells following co-culture with *H. pylori* strain 26695 for 24 h was measured by an ELISA assay (R&D systems, Minneapolis, USA).

Anti-iNOS (Santa Cruz Biotech, 1∶200), anti-COX-2 (Neomarkers, 1∶200), and anti-α-tubulin (Sigma, 1∶10000) as a loading control were used for Western blot analysis of protein extracts from AGS cells infected with *H. pylori* strain 26695. Briefly, cells were lysed in cold Catenin lysis buffer 1% Triton X-100 (Sigma) in PBS enriched with a protease inhibitor cocktail (Roche) and a phosphatase inhibitor cocktail (Sigma). Proteins were quantified using a modified Bradford assay (Bio-Rad). Thirty-µg of proteins were loaded on a 7.5% or 12% SDS–PAGE. Donkey anti-rabbit (Amersham Biosciences) or sheep anti-mouse (Amersham Biosciences) HRP-conjugated secondary antibodies were used. Immunoblots were revealed by ECL detection (Amersham Biosciences) and quantified using the Quantity One Software (Bio-Rad).

### Analysis of components of bacterial cell wall

To analyze the effect of DHA on the composition of the outer membrane proteins of *H. pylori*, membrane protein extracts were prepared from 200 ml of strain 26695 12 h culture incubated with 100 or 250 µM of DHA or in absence of DHA. Bacteria were sonicated in 10 mM Tris-HCl, pH 8.0. After two centrifugations for 20 min at 5500 rpm, the supernatants containing the membrane proteins were centrifuged at 37500 rpm for 1 h. The pellet was resuspended in 40 µL solubilisation buffer (10 mM Tris-HCl pH 7.5, 7 mM EDTA, 0.6% sarcosyl). After a second ultracentrifugation step for 2 h, the pellet containing the outer membrane fraction was resuspended in 25 µL of 10 mM Tris-HCl, pH 8.0. Proteins were then separated by 6% SDS-PAGE and stained with Coomassie Brilliant Blue [21].

The outer membrane protein composition of *H. pylori* cell envelop was also analyzed by 2D electrophoresis gel. Briefly, 150 µg of proteins prepared as above, were precipitated accordingly to the manufacturer protocol (ProteoExtract, Calbiochem). Proteins were then resolubilized in a 2-D rehydration buffer (DeStreak, GE Healthcare), containing 0.2% ampholytes (Bio-rad) and then loaded overnight by passive rehydration onto a 3–10 non-linear pH IPG (immobilized pH gradient) strips (Biorad). In the first dimension, proteins were separated by isoelectric focusing (IEF) using a three steps method at 20°C constant temperature and a current limitation of 50 microA/strip: step 1, 0–250 V for 15 min (rapid ramp); step 2, 250–4000 V for 2 h (rapid ramp); and step 3, constant 4000 V until reach, approximately, a total of 15000 V-hr. After IEF, strips were equilibrated in a reducing equilibration buffer (6 M urea, 0.375 M Tris-HCl pH 8.8, 20% (v/v) glycerol, 2% (v/v) SDS, 0.002% (w/v) bromophenol blue and 2% (w/v) DTT) for 10–15 min followed by incubation with an alkylating equilibration buffer, for 10–15 min, with the same composition but with iodoacetamide replacing DTT at a 2.5% (w/v) concentration. For the second dimension analysis, the strips were washed with electrophoresis buffer and fixed on the surface of a commercial precast gel with a 4–20% gradient (Mini-Protean TGX IPG, Bio-rad) in order to perform the protein separation by SDS-PAGE electrophoresis. The electrical current conditions started at 8 mA/gel until 24 mA/gel with a maximum of 200 V until the dye reached the bottom of the gel. Furthermore, protein gels were Coomassie Blue stained using a commercial solution (PageBlue, Coomassie Brilliant Blue G-250, Thermo), following manufacturer's instructions.

MreC expression was assessed by Western blot analysis on *H. pylori* extracts from strain 26695 cultured until 1 OD600 using a rabbit anti-MreC antibody (1∶10000) [22]. Β-Urease was used as a loading control.

*H. pylori* LPS was prepared from strains 26695 and SS1 grown for 12 h without DHA (control) or with 100 µM of DHA, as previously described [23], separated by (SDS)-polyacrylamide gel electrophoresis [24] and visualized by silver staining [25].

### Image analysis

The stained 2-DE gels were scanned with a calibrated densitometer (GS-800, Biorad) with 36.3×36.3 (X×Y) microns resolution, 700 dpi. The spot 2D-detection, background removal and matching were performed by the PDQuest software v8.01 (Bio-Rad). In order to quantify and to compare the individual proteins spots, all the gel images were normalized using a local regression model. Three biological replicates with four gels were performed for each control and DHA treated samples. For this comparative analysis, only the spots present in every replicate were considered. Proteins were classified as being differentially expressed in DHA treated samples when spot intensity showed a difference higher than 1.4 fold in comparison to the untreated control. All the matching spots were rechecked manually. Significant differences in protein expression levels were determined by different statistical tests: Student's t-test with a set value of p<0.05, Mann-Whitney signed rank test and partial least squares test.

### Protein Identification by MALDI TOF/TOF mass spectrometry

The selected protein gel spots were processed for protein identification in accordance to the trypsin manufacturer recommended protocol (Promega). Protein digests were desalted, concentrated and spotted onto a MALDI plate using C18 reverse phase chromatography (ZipTips, Millipore). For the matrix, a solution of 6–7 mg/mL alpha-cyano-4-hydroxycinnamic acid in 50% ACN/0.1% TFA was used. Samples were analyzed using a 4700 Proteomics Analyzer MALDI-TOF/TOF (AB SCIEX, USA) by Peptide Mass Fingerprint (PMF) and, whenever necessary, by MS/MS peptide sequencing as previously described [26].

The identification of the proteins was done by search in Swiss-Prot and TrEMBL, of the UniProtKB protein sequence knowledgebase (release 2011_12) using the Mascot search engine (Version 2.1.04) with the following modifications: constant - carbamidomethylation (Cys) and variable - oxidation (Met).

### Statistical Analysis

Data are expressed as means ± standard deviations (SD) whenever data follows a normal distribution. The significance of differences between experimental data was validated using Student's *t*-test. All statistical tests were two-sided. Differences were considered significant when *P*<0.05.

## Results

### DHA inhibitory effect on *H. pylori* growth

**Figure 1:**
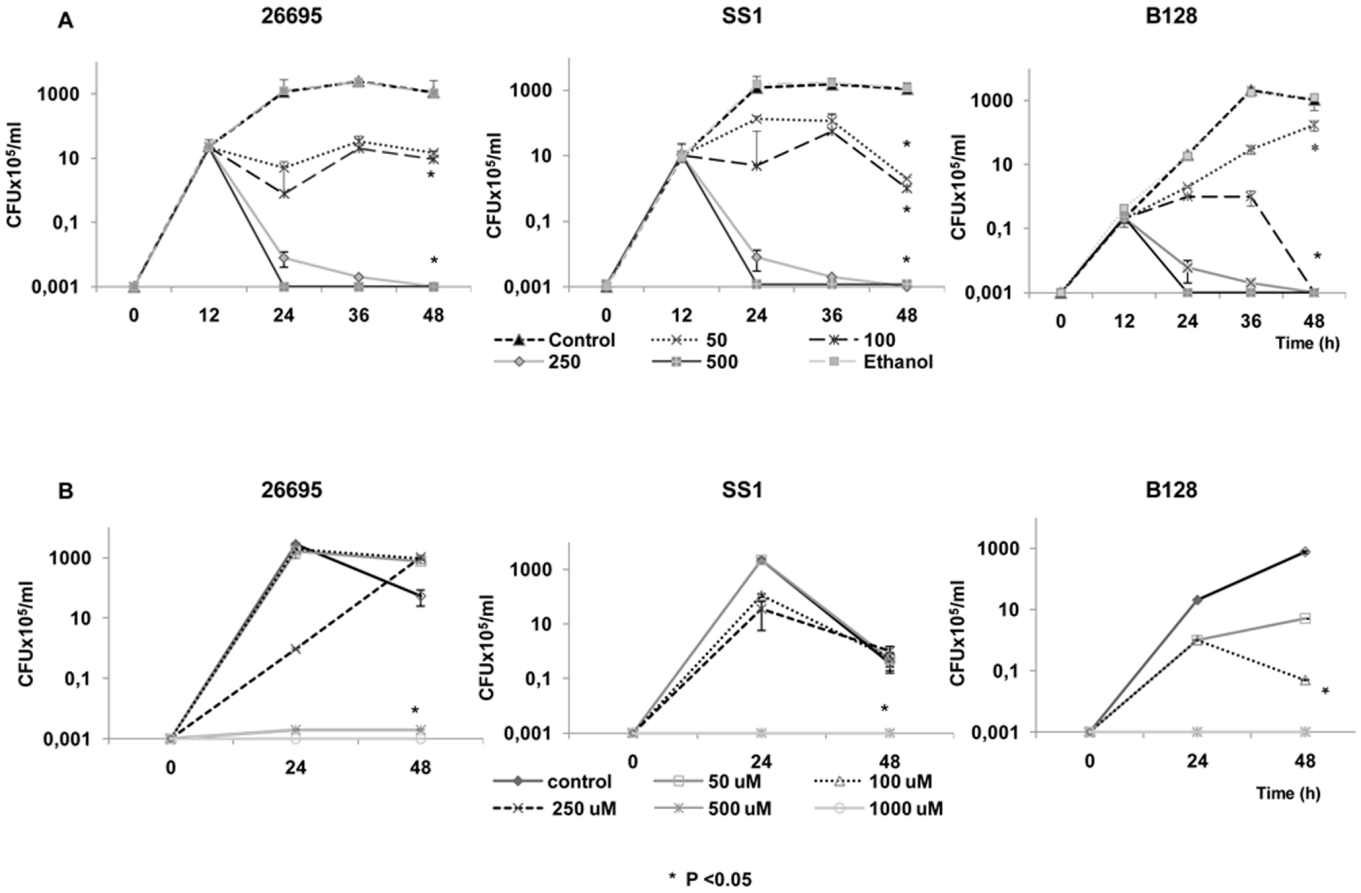
Growth of *H. pylori* in the presence of increasing concentrations of DHA. A) DHA was added to the *H. pylori* culture strains 26695, SS1 and B128 after 12 h of growth. Each experiment, consisting of a control (non-treated *H. pylori* culture) and *H. pylori* incubated with DHA (concentrations of 50 µM, 100 µM, 250 µM and 500 µM) performed in triplicate. Ethanol (0.06% v/v) the solvent of DHA stock solutions was also tested in these experiments and it did not influence *H. pylori* growth. B) *H. pylori* was grown in liquid cultures with concentrations of DHA ranging from 50 µM to 1000 µM. Twenty-four hours later cells were pellet and put to grow into a fresh medium without DHA. *H. pylori* growth was irreversibly inhibited by DHA at the highest concentrations (≥500 µM of DHA). Data are expressed as the mean ± Standard Deviation and are representative of three independent experiments. * Refers to significant differences in *H. pylori* growth between controls and DHA-treated conditions (50 µM to 1000 µM of DHA).

*H. pylori* strains 26695, SS1 and B128 were grown for 12 h in liquid culture and then treated with DHA at concentrations of 50 µM, 100 µM, 250 µM and 500 µM. Concentrations higher than 100 µM of DHA significantly reduced *H. pylori* growth (P<0.05), both in terms of optical density (results not shown) and CFU. These results confirm that DHA has an inhibitory effect on *H. pylori* growth (Figure 1A).

In an attempt to assess if DHA effect on *H. pylori* growth was irreversible, bacterial cells were cultured in the presence of increasing concentrations of DHA from 50 µM to 1000 µM during 24 h as previously described. Then, cultures were harvested, washed and bacteria let to grow in DHA free fresh medium. As shown in Figure 1B, *H*. *pylori* previously treated with 50 µM, 100 µM or 250 µM of DHA was able to survive in a DHA-free medium. However, concentrations higher than 500 µM of DHA caused irreversible effects on *H. pylori*, leading to growth arrest under the same conditions. All three strains grew similarly in the presence of DHA.

**Figure 2:**
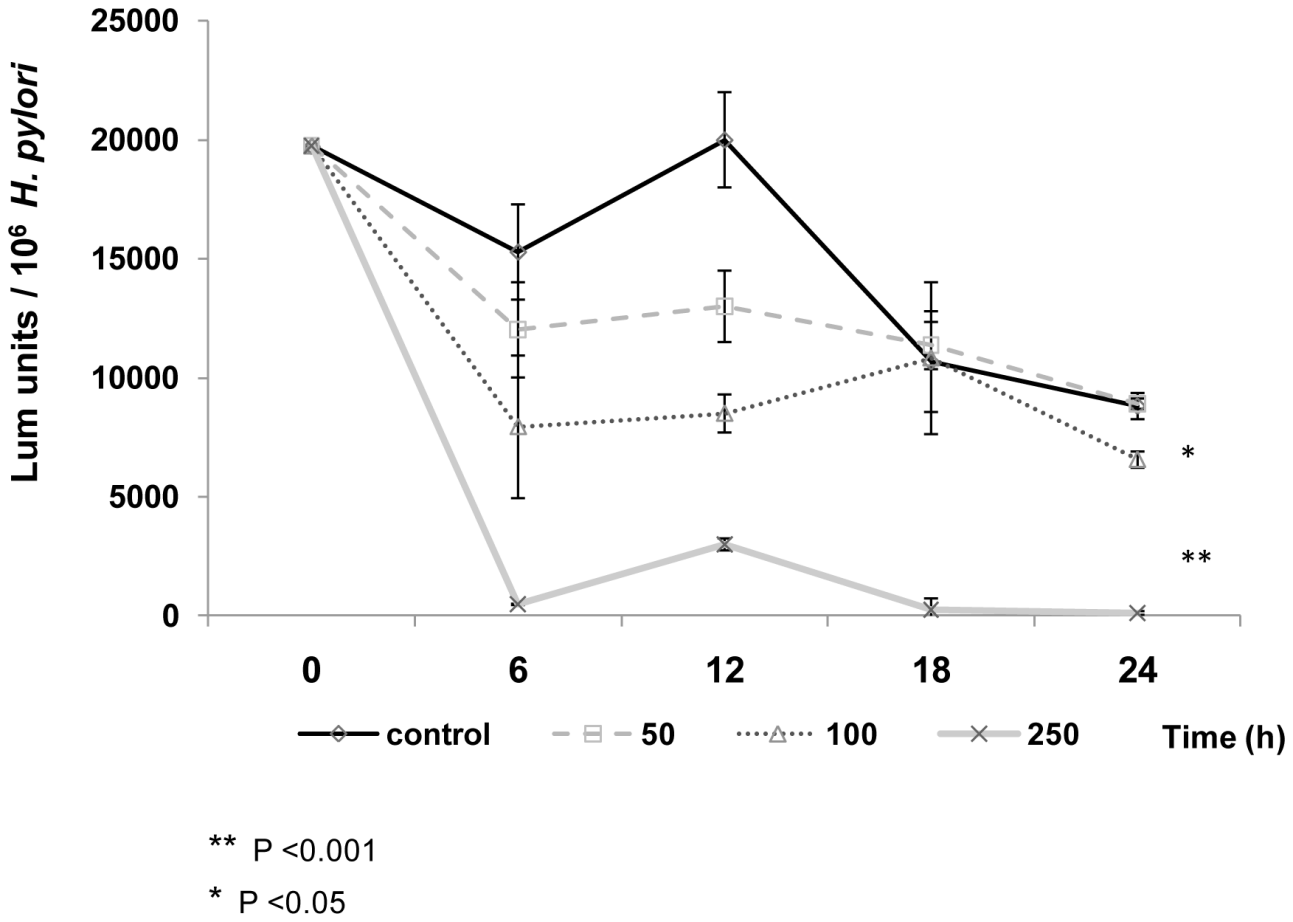
DHA impairs *H. pylori m*etabolic activity. Cultures of *H. pylori* 26695 grew for 24 h, and every six hours the relative ATP levels were measured as described in materials and methods. Luminescence units per 10^6^ bacteria at each time point resulted from 3 independent experiments. *T-test* analysis confirmed a significant differences in ATP production between *H. pylori* untreated controls and *H. pylori* treated with 100 µM of DHA, (P<0.05) and with 250 µM of DHA (P<0.001).

To investigate if DHA inhibitory effect on *H. pylori* growth could be associated with alterations of the general metabolic activity of the bacteria, we also analyzed ATP production over time in the *H. pylori* strain 26695 (Figure 2). Concentrations higher than 100 µM of DHA significantly reduced *H. pylori* ATP production, compared to controls, indicating that DHA also impairs the metabolism of bacteria.

### DHA treatment decreases *H. pylori* adhesion to gastric epithelial cells and reduces inflammation

**Figure 3:**
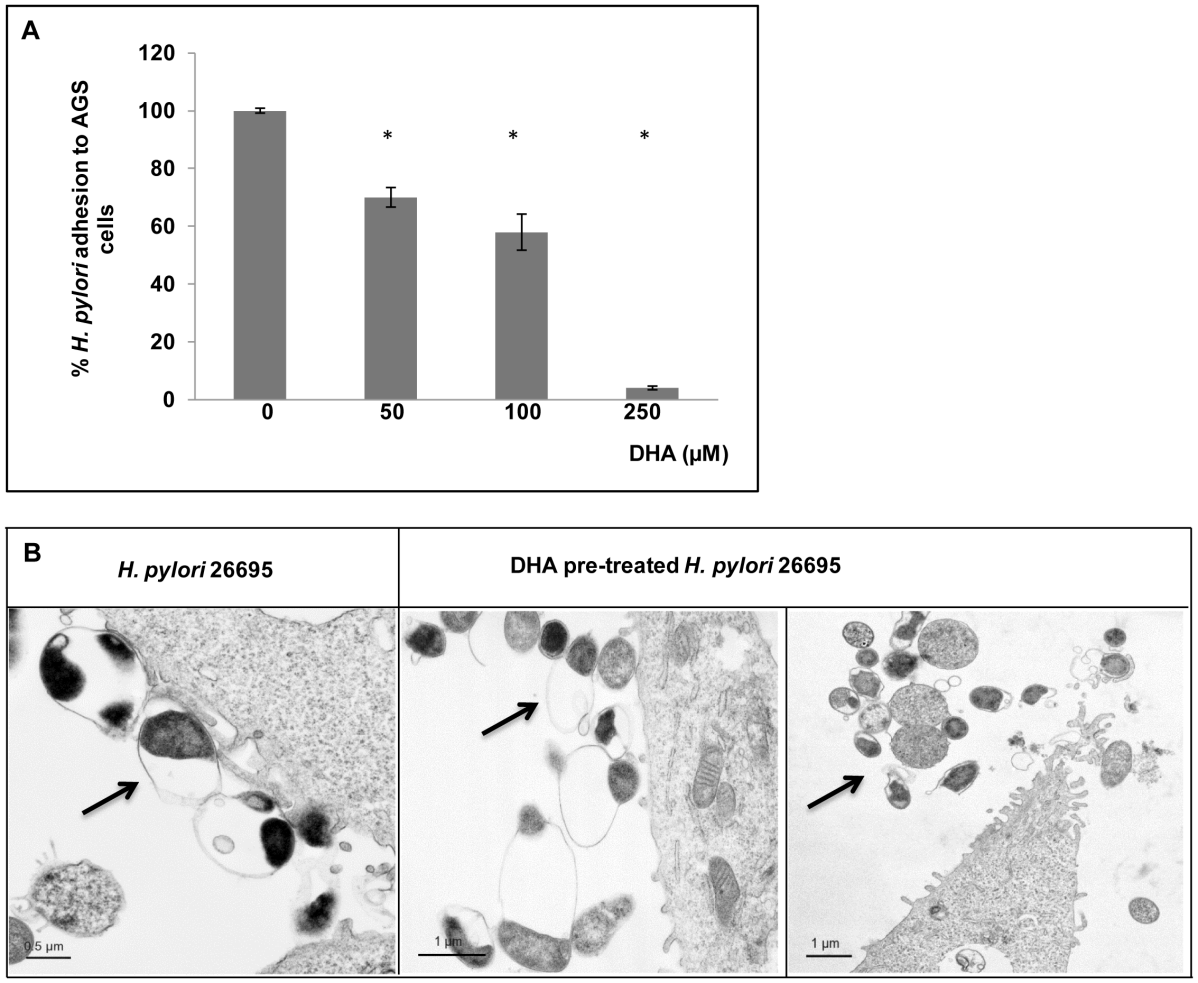
DHA treatment decreases *H. pylori* adhesion to epithelial gastric cells. AGS cells are infected 24 h either with *H. pylori* strain 26695 pre-treated for 24 h with DHA 50 µM, 100 µM or 250 µM, or non-treated bacteria. A) Quantification of *H. pylori* attachment to AGS cells by ELISA using and anti-*Helicobacter* antibody. Pretreatment of *H. pylori* with DHA significantly decreased the bacterial adherence to AGS cells for treatment of 50 µM and above (*P<0.05). Data are expressed as the mean ± SE and are representative of three independent experiments. B) TEM observation of cells shows less attachment of *H. pylori* DHA – pretreated (100 µM) compared to cells infected with non-treated bacteria.

Bacterial attachment to gastric epithelial cells plays a central role in *H. pylori* colonization and efficient delivery of virulence factors. In order to analyze if DHA alters bacteria attachment to epithelial cells, AGS cells were incubated 30 min either with *H. pylori* strain 26695 previously treated for 24 h with increasing concentrations of DHA or with non-treated bacteria. The adherence of bacteria was quantified using an anti-*Helicobacter* antibody by ELISA. As reported in Figure 3A, the percentage of bacterial adhesion to epithelial cells decreased significantly when bacteria were pre-treated with DHA. Adhesion was completely inhibited at the highest concentration of 250 µM. These data were confirmed by TEM observation of co-cultures performed under the same conditions. Pre-treatment of bacteria with 100 µM of DHA led to a lower number of bacteria attached to cells compared to cells infected with non-treated bacteria (Figure 3B).

**Figure 4:**
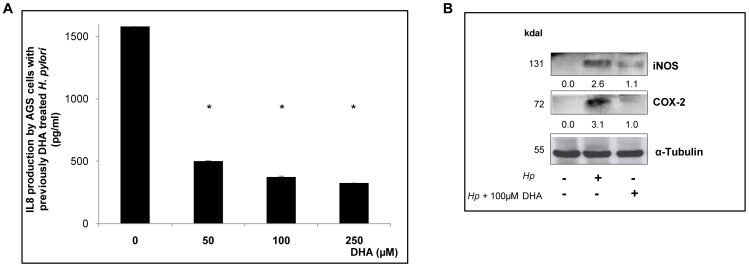
DHA pre-treatment of *H. pylori* leads to a reduced inflammatory response. A) AGS cells were cocultured with *H. pylori* strain 26695 for 24 h previously exposed to DHA, and then measured IL-8 production with an ELISA-like test. Pre-treatment of bacteria with DHA from 50 µM to 250 µM led to a significant decrease of 4-fold in IL-8 production as compared to non-DHA treated bacteria * P<0.05. Data are expressed as the mean ± SE and are representative of three independent experiments. B) Expression of COX2 and iNOS on protein extracts from AGS cells infected with *H. pylori* 26695 previously treated with DHA. α-tubulin corresponds to the loading control. The values are reported under the corresponding gel bands and showed a significant decrease of COX2, and iNOS levels in cells infected with DHA-pretreated *H. pylori* compared to untreated bacteria.

We next assessed the consequences of DHA exposure to the inflammatory response of gastric epithelial cells infected by *H. pylori*. IL-8 is a chemokine responsible for the acute active inflammation characterized by neutrophils infiltration and induction of inflammatory mediators. Il-8 is increased in the gastric mucosa of *H. pylori* infected patients and in the gastric epithelial cells inoculated *in vitro* [27]. The IL-8 production was measured in AGS cells co-cultured with DHA pre-treated *H. pylori* strain 26695 for 24 h, under the same conditions as described above (Figure 4A). Pre-treatment of bacteria with DHA from 50 µM to 250 µM led to a significant decrease of 3 to 4-fold in IL-8 production as compared to cells infected with untreated bacteria (P<0.05). In line with this, immunoblot analysis showed that protein levels of important inflammatory mediators, such as COX-2 and iNOS were significantly decreased (2 to 3 fold) in cells infected with *H. pylori* previously treated with DHA 100 µM (P<0.05) (Figure 4B).

### DHA induces changes in the protein composition of *H. pylori* membrane and cell wall

**Figure 5:**
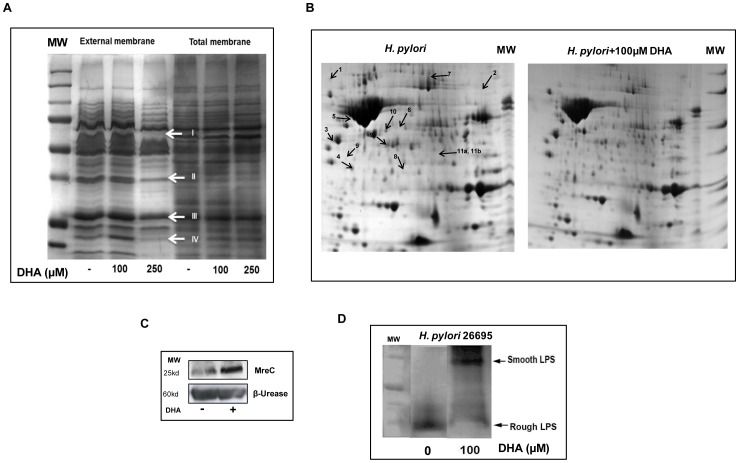
Consequences of DHA exposure on *H. pylori* protein profile, LPS phenotype and MreC expression. A) *H. pylori* 26695 proteins profile from outer membrane extracts in a 1D SDS-PAGE gel. Arrows are pointing out five bands that correspond to proteins with a decreased level after treatment of bacteria with 100 µM and 250 µM of DHA. Candidate proteins were identified and referred as FrpB4/HP1512 (arrow I), HorL/HP1395 (arrow II), HorB/HP0127 (arrow III), and HorJ/HP1469 (arrow IV). B) *H. pylori* 26695 proteins profile for outer membrane extracts in a 2D SDS-PAGE gel. Twelve proteins identified by MALDI TOF/TOF mass spectrometry presented a differential expression level when comparing untreated control and DHA-treated bacteria. These proteins were mostly related with induction of stress condition as listed in the Table 1. C) Western blot analysis of MreC protein level in DHA-treated bacteria. *H. pylori* 26695 grew as previously described until a maximum of 1 OD600. MreC expression was assessed using a rabbit anti-MreC antibody (1∶10000) [22]. D) *H. pylori* 26695 LPS profile. LPS was extracted from *H. pylori* strain 26695 cultured for 12 h in the presence of 100 µM of DHA and silver stained in a Trycine SDS-PAGE as described in material and methods. In absence of DHA, the *H. pylori* strain 26695 showed a low molecular weight rough (R)-form LPS phenotype. At concentrations of 100 µM of DHA, the LPS shifted from (R)-form to the high molecular weight smooth (S) form phenotype.

The *H. pylori* morphological changes induced by DHA previously described [17] associated with the impairment of bacterial metabolism and the inhibitory effect of DHA on bacteria cell adhesion led us to postulate that this molecule should alter the bacterial membrane proteins composition and cell wall. Figure 5A represents 1D-gel electrophoresis of *H. pylori* total and outer membrane protein extracts. Protein patterns corresponding to total membrane extract did not vary significantly in the presence of DHA. However, the outer membrane protein extracts corresponding to bacteria exposed to 100 µM of DHA, a condition that reduces *H. pylori* growth (Figure 1A), showed variations of the pattern of bands (marked by arrows on Figure 5A). Mass spectrometry analysis was conducted to identify proteins with altered expression. These candidate proteins were referred as FrpB4/HP1512 a nickel TonB-dependent transporter [28] (arrow I) and three other outer membrane proteins of the same paralog family HorJ/HP1469 (arrow II), HorB/HP0127 (arrow III), and HorL/HP1395 (arrow IV). For some of them, the estimated molecular weight (MW) was lower than the calculated MW and those bands could correspond to degradation products.

To further identify the proteins which presented variation in the expression pattern due to DHA treatment on the outer membrane protein composition, a 2D-electrophoresis gel was performed under the same conditions *i.e* treatment of bacteria with DHA 100 µM as previously described (Figure 5B). Treatment of *H. pylori* with DHA induced changes in the outer membrane proteins listed in Table 1. Twelve outer membrane proteins as highlighted on the gel (Figure 5B), showed differential levels in DHA-treated compared to untreated bacteria. Expression of heat shock proteins (HP0515), chaperones (Dnak; HP0109) and proteins involved in response to oxidative and various stress conditions, such as Catalase (HP0875), Urease accessory protein UreG (HP0068) and UreH (HP0067), *Gamma-*glutamyltranspeptidase (γGT) (HP1118) and Non-heme iron containing Ferritin (Pfr) (HP0653) were deregulated. N-methylhydantoinase (HP0696), responsible for detoxification of acetone and its use as an additional carbon source under nutrient-limiting conditions [29], was significantly decreased (P<0.05). The actin-like MreB protein (HP1373) affects the progression of the cell cycle [30] and was increased in DHA-treated *H. pylori*. Since the *mreB* and *mreC* genes are part of the same operon and because MreC (HP1372) is a rod-shape determining protein [22] we also evaluated whether there were changes in the expression of this protein by DHA. As observed for MreB, MreC level was also increased in *H. pylori* treated with DHA (Figure 5C). Another important protein characterized as a virulence factor for *H. pylori*, the Neutrophil-activating protein A (Hp-NapA) (HP0243) known to be involved in cell adhesion is increased upon DHA treatment. It binds to mucins Lewis X blood-group antigens. Hp-NapA acts as a Th1-stimulating antigen in humans and it has been reported to activate neutrophil granulocytes *in vitro* [31], [32]. It contributes to the ability of *H. pylori* to survive to oxidative stress. Overall, these data suggest that DHA exposure induces damage associated to stress conditions in bacteria as indicated by the genes signature profile observed in response to DHA effect.

**Table 1:** Identified *H. pylori* outer membrane proteins differentially expressed following DHA treatment.

No	Protein	MW (pI)§	Protein score C.I.%	Mascot score	Distinct peptides	% coverage	Variation upon DHA; *p* value
1	Chaperone DnaK (HP0109)	67.1 (5.0)	100	356	37	55	↑1.6x; p<0.05
2	Catalase (HP0875)	58.7 (8.7)	100	341	37	63	↓0.6x; p<0.05
3	Urease accessory protein ureG (HP0068)	22.1 (5.03)	95	66	9	43	↑2.7x; p<0.05
4	HP-NAP (HP0243)	16.9 (5.6)	100	156	16	75	↑2.5x; p<0.05
5	Rod shape (HP1373)	37.5 (5.4)	100	183	21	55	↑2.2x; p<0.05
6							
	Methionine aminopeptidase (HP1299)	27.8 (6.0)	99.9	85	11	41	↑2.0x; p<0.05
7	Methylhydantoinase (HP0696)	87 (6.49)	100	246	33	35	↓0.6x; p<0.05
8	Heat shock protein A (HP 0011)	12.9 (6.1)	99.6	78	9	52	↑1.6x; p<0.05
9	Ferritin (HP0653)	19.3 (5.4)	100	296	6	53	↑1.4x; p<0.05
10	Urease accessory protein ureH (HP0067)	30 (5.8)	100	119	14	60	↑1.4x; p<0.05
11a*	γ-glutamyl-transpeptidase (HP1118)	61 (9.3)	100	167	11	15	↓0.51x; p<0.05
11b*	ATP-dependent protease HslV (HP0515)	20 (6.5)	99.9	97	3	19	↓0.51x; p<0.05

§ *Molecular weight in kDa and pI in parentheses. Spot Mascot legend depicted in* *figure 5B* *.*

* Proteins 17a and 17b were identified from the same spot in the 2D-electrophoresis gel. Mascot score is defined as −10*Log10 (P), where P is the absolute probability. Protein score confidence interval (C.I.) % rates the confidence level of the Mascot Score to a 5% significance level. The closer the C.I.% is to 100%, the more likely the protein is correctly identified.

Twelve proteins identified by MALDI TOF/TOF mass spectrometry presented a differential expression level when comparing untreated control and DHA-treated bacteria. These proteins were mostly related with induction of stress condition.

In addition to membrane protein profiles, we also evaluated the impact of DHA on peptidoglycan (PG) and LPS structures, important components of bacterial cell wall also considered as virulence factors [33], [34]. While the PG composition did not change significantly in DHA treated bacteria (data not shown), the *H. pylori* 26695 LPS was modified from rough to smooth phenotype in DHA-treated bacteria compared to untreated *H. pylori* (Figure 5D).

## Discussion

In a previous study we identified DHA as a novel antibacterial agent with anti-*H. pylori* activity *in vitro and in vivo*. Indeed, our data also demonstrated that *H. pylori* infected mice that received DHA-supplemented drinking water, presented 50% lower gastric mucosa colonization levels and lower severity of gastritis, compared to untreated mice [17]. Our present study further investigates the action of DHA to identify the bacterial components at the origin of its anti-*H. pylori* effect as well as to define the impact on host inflammatory response to infection. Associated with the alteration of bacteria morphology previously shown upon DHA exposure [17], we demonstrated that DHA modifies the *H. pylori* outer membrane protein composition associated with the induction of a general stress condition and alteration of bacterial metabolism. These DHA effects lead to the inhibition of *H. pylori* adherence to host epithelial cells and of its ability to induce the inflammatory host response.

In the present study, DHA was found to inhibit *H. pylori* growth *in vitro* when added in the log phase to a bacteria liquid culture. DHA leads to an inhibition of viable colonies at the highest concentration of 250 µM, supporting our previous results [17]. Nevertheless, the effect of DHA was found to be reversible until a concentration of 250 µM. Hence, after exposure to DHA, *H. pylori* bacteria transferred to a DHA-free medium recovered and grew. On the other hand, a previous treatment of *H. pylori* with a concentration of DHA higher than 500 µM causes irreversible effects and arrest growth. This indicates that in order to exert an optimal inhibitory action on *H. pylori* growth, bacteria needs to be continuously exposed to DHA. The ability to recover from DHA treatment might explain our results concerning *H. pylori* gastric colonization in mice treated with DHA, which showed that although exposure to DHA was continuous, in some mice *H. pylori* grew normally and were able to recover from DHA-induced damage [17].

Our findings also suggest that exposure to DHA affects the outer membrane protein composition with a reduction of ATP production by bacteria. In fact, ATP synthesis, which is known to decrease with anti-inflammatory molecules, relies on the electron respiratory chain that might be altered by DHA's effect on *H. pylori* membranes [35]. Moreover, mass spectrometry analysis identified changes in *H. pylori* proteins related with a general stress response induced by DHA. Changes in the expression of *H. pylori* heat shock proteins, chaperones, and proteins involved in oxidative stress response like catalase and Hp-NapA were observed. Decrease in catalase (HP0875) and γ*GT* expression may cause a lower ability of the bacteria to neutralize hydrogen peroxide and to respond to stress conditions respectively. *H. pylori* γGT is also essential for nitrogen metabolism in *H. pylori* [36]. Therefore, the observed decrease of *H. pylori* γGT is unlikely to be related to reduced bacterial defences against ROS, but more probably associated with *H. pylori* metabolism [37]–[40]. Non-heme iron containing Ferritin (Pfr) (HP0653), which serves as an intracellular iron storage, protects *H. pylori* against iron starvation and from acid-amplified iron toxicity [41], was shown to be increased upon DHA treatment. Additionally, iron storage and homeostasis is essential for *H. pylori* to survive in its hostile natural environment [41], [42]. Two urease accessory proteins, UreG and UreH involved in Nickel incorporation into urease are also induced in the presence of DHA. It is interesting to notice that our previous study [21] on the *in vivo* interactome of *H. pylori* reported an interaction between UreG and two iron storage proteins also induced by DHA, Frp and NapA. This deregulation of the expression of *H. pylori* outer membrane proteins might be related to the decrease of *H. pylori* adhesion to host epithelial cells, leading to the inhibition of bacterial colonization in mice stomach [17]. M*reC* has been identified as one of the responsible proteins for confining *H. pylori* spherical shape, suggestive of a close involvement of this protein in *H. pylori* rod-shape maintenance [22], [30]. As reported in the literature, a decrease in MreB and MreC levels is associated to changes in *H. pylori* morphology (from rod to spherical) [22]. Our data lead us to postulate that increase of MreB and MreC observed in bacteria exposed to DHA results from a feedback regulation in an attempt to compensate its effect on bacteria cell wall, leading to an overexpression of the proteins needed to circumvent the damage imposed by DHA. Finally, DHA treatment induced a change in the LPS profile from rough to smooth. A smooth LPS presents a polymerized O-antigen creating an additional polysaccharide barrier on the bacterial surface, as a potential strategy for bacteria to enhance resistance to DHA diffusion.

Our study shows that DHA inhibits the response of important players in *H. pylori* mediated inflammation, such as iNOS and COX-2 expression [43]. Accordingly, cells infected with DHA pre-treated *H. pylori* showed a decrease of COX-2 and iNOS expression. COX-2 is responsible for the production of prostaglandin-E_2_ [44], previously shown to be decreased in the serum of *H. pylori* infected mice treated with DHA [17]. In agreement with our results, an inhibition of COX-2 and iNOS levels by n-3 PUFAs confirms the anti-inflammatory role of these lipids as extensively reported elsewhere [45]–[47].

During our previous experiments in the mouse model [17], we assumed that gastric pH did not vary significantly, since DHA is a weak acid and therefore its likelihood to dissociate and release H^+^ ions into solutions is very low, moreover in very acidic gastric settings as the human stomach. Nevertheless, DHA affects cell metabolism and intracellular pH, most probably through diffusion into bacterial cell wall, leading to increased permeability and acidity within bacterium [48], [49]. A pertinent question would be if DHA is able to affect antibiotics efficiency in *H. pylori* treatment through pH modulation. Petschow *et al* have shown that fatty acids that hold an *in vitro* anti-*H. pylori* growth effect do not develop resistance spontaneous and, therefore, do not pose concern regarding *H. pylori* antibiotics resistant strains [50]. In the same line, we have shown that DHA decreases recurrence of infection in mice two months after being treated with a standard 7-days antibiotic treatment (amoxicillin, metronidazole and omeprazole) [17]. Omeprazole included in the antibiotherapy is a proton pump inhibitor that increases the local pH to optimize the action of antibiotics. In line of this, presence of omeprazole should also have the same effect on DHA activity. The acidic gastric pH (pH 2.0) does not seem to affect the chemical stability of the antibiotics usually prescribed for *H. pylori* infection treatment [51]. In this context we cannot exclude a synergistic action between DHA and antibiotics, possibly through an increase in the exposure of the bacterium to antibiotic molecules in the gastric milieu or an enhancement of their delivery. Nevertheless, the mechanisms should not include pH as a crucial determinant since DHA is unlikely to alter pH values in the gastric environment.

In conclusion, our results demonstrate that DHA impairs *H. pylori* survival through several mechanisms that include the alteration of *H. pylori* outer membrane protein composition related to alteration of bacteria morphology [17], the induction of a general stress response, and the modulation of bacteria metabolism that altogether contribute to decrease *H. pylori* adhesion to gastric epithelial cells and to limit its inflammatory effect. This study reports important aspects of the mechanisms responsible for anti-*H. pylori* effects of DHA including the reduction of inflammation and the arrest of *H. pylori* growth. Overall, our results further support the use of DHA as an adjuvant in anti-*H. pylori* treatments promoting the efficacy of standard antibiotherapy as suggested by our previous data in the mouse model [17].
